# Virtual reality and annotated radiological data as effective and motivating tools to help Social Sciences students learn neuroanatomy

**DOI:** 10.1038/s41598-021-92109-y

**Published:** 2021-06-18

**Authors:** Margot van Deursen, Laura Reuvers, Jacobus Dylan Duits, Guido de Jong, Marianne van den Hurk, Dylan Henssen

**Affiliations:** 1 Department of Educational Sciences, Faculty of Social Sciences, Radboud University, Nijmegen, The Netherlands; 2 Department of Medical Imaging, Radboud University Medical Center, Nijmegen, The Netherlands; 3 Radboudumc 3D Lab, Radboud University Medical Center, Nijmegen, The Netherlands

**Keywords:** Anatomy, Nervous system, Brain

## Abstract

Neuroanatomy as a subject is important to learn, because a good understanding of neuroanatomy supports the establishment of a correct diagnosis in neurological patients. However, rapid changes in curricula reduced time assigned to study (neuro)anatomy. Therefore, it is important to find alternative teaching methods to study the complex three-dimensional structure of the brain. The aim of this manuscript was to explore the effectiveness of Virtual Reality (VR) in comparison with Radiological Data (RaD) as suitable learning methods to build knowledge and increase motivation for learning neuroanatomy. Forty-seven students (mean age of 19.47 ± 0.54 years; 43 females; 4 males) were included; 23 students comprised the VR group. Both methods showed to improve knowledge significantly, the improvement between groups was not different. The RaD group showed to have a significantly higher score on expectancy than students in the VR group. Task value scores regarding finding a task interesting, useful and fun were found to be significantly different in favor of the VR group. Consequently, significant higher Motivation scores were found in the VR group. Motivation and expectancy, however, did not moderate learning results, whereas task value impacted the results in favour of the VR group. This study concludes that VR and RaD are effective and diverting methods to learn neuroanatomy, with VR being more motivating than RaD. Future research should investigate motivation and task value when using VR over a longer period of time.

## Introduction

Over recent years, medical and psychology curricula have changed drastically, resulting in a renewed focus on communication skills and less time devoted to study anatomy^1–4^. Especially the subspecialty of neuroanatomy suffers from these reductions considering that students find it difficult to understand the three-dimensional (3D) relations of different brain structures^5^. Traditionally, neuroanatomy in medical and psychology curricula is learned by lectures and a combination of brain dissection, the use of (plastinated) prosections, plastic anatomical models and studying neuroanatomical atlases^5–10^. Furthermore, some faculties have described to use brightly colored clay to help students learn neuroanatomical structures^11,12^.

In order to optimize learning during the sparse hours of neuroanatomy education, technological innovations have been suggested as a valuable addition to other neuroanatomy education tools. These technological innovations include various forms of electronic 3D models^13–15^. Generally, these electronic 3D models are based on consecutively stacked two-dimensional (2D) data, including radiological imaging data (e.g., magnetic resonance imaging; MRI, computed tomography; CT). Incorporating screen-based radiological data (RaD) in anatomy education has been reported frequently in literature.

The use of post-processed data in the form of electronic 3D models comprises various options, including virtual reality (VR) models. VR was introduced in the 1960’s and is defined as a computer-generated simulation of a 3D environment that can be interacted with in a seemingly real or physical way by the user^16^. One of the advantages of VR is that it provides the opportunity to practice situations that are potentially dangerous to practice in real life or cannot be experienced physically (e.g., physical inaccessibility). The use of VR has been suggested to be a valuable asset for a broad variety of challenges, including treatment of gambling addiction^17^ and depression^18^, neurorehabilitation^19,20^ and pain reduction^21^. In medical educational research, VR has been reported to have a positive effect on learning abilities^22–25^. In anatomy education, VR environments can be implemented as less expensive and promising alternative to cadaver dissection^26^. Additionally, VR has been reported as an effective learning tool, especially for students with lower spatial abilities^25,27,28^. Furthermore, the use of VR in anatomy education promotes intrinsic benefits such as increased learner immersion and engagement^29–32^.

With regard to RaD, most medical curricula are known to use imaging data in their anatomy education programs. These teaching approaches have steadily developed since the 1970s when the first reports on the use of X-rays in anatomy education were published^33^. Nowadays, the vast majority of medical curricula have integrated some sort of radiological imaging into their anatomy education^34–36^. Over the years, integration of clinical images obtained from high-resolution CT and MRI scans has been described in literature as well (for an overview, see^37^). The implementation of these imaging data in anatomy education has propelled the development of RaD in the form of online radiology atlases with labeled radiology data^38,39^. It has been reported that the integration of RaD in anatomy education allows students to acquire a spatial understanding of anatomical structures and their complex relative positions^40–43^. However, the implementation of RaD features in psychology education remains understudied.

Both the use of RaD and VR with 3D models are considered interactive multimodal learning environments as at least two different modes are used to represent the learning content^44^. Multimodal learning environments can be interactive and non-interactive^44^. Classification of the different types of interactivity concern dialoguing, controlling, manipulating, searching and navigating. Both RaD and VR can be considered as manipulating interactive modalities as the learner can use the learning tool to zoom in or out, move objects around and interact in other ways with virtual data^44^. Next to determining whether a tool serves non-interactive or interactive learning, a distinction can be made between two views of learning: information acquisition and knowledge construction^45^. With information acquisition learning involves adding information to the learner’s memory. The learner’s job is to receive information. The most fitting learning environment for information acquisition is a non-interactive environment. Knowledge construction, on the other hand, involves building a mental representation. The learner’s job here is to select, organize and integrate new information with existing knowledge. The goal of the instruction method is to guide the learner to actively fathom the instructional materials^46^. The most fitting learning environment for knowledge construction is an interactive multimodal learning environment^47^ as incorporating interactivity can promote deeper learning from a multimedia explanation if it is done in a theory-based way. Thereby, both RaD and VR are hypothesized to be effective methods to help students learn (neuro)anatomy.

Interactive multimodal learning environments such as VR and RaD also motivate learners to engage in the cognitive processes^48^. Because of this, when assessing the value of a new teaching method, it is important to consider their effect on student motivation. Moreno and Mayer (2007) support this statement by stating that motivational factors mediate learning by increasing or decreasing cognitive engagement^44^. Parong and Mayer reported that a lesson on scientific information in a VR environment was experienced as more enjoyable and motivating as compared to an equivalent lesson in a PowerPoint slideshow^49^. In turn, motivation is known to result in better quality of learning^50^. Individuals’ expectancies for success on a specific task and the value of the task are other important determinants of their motivation on achievement tasks^51^. The expectancy-value theory has been one of the most important views on the nature of achievement motivation. Thereby, a relationship can be identified between competence beliefs and expectancies for success (for an extensive overview see^51^). However, to which extend these educational and motivational theories actually play a role in (neuro)anatomy education, and especially the use of RaD and VR with 3D models, has not been investigated before.

The current study therefore aimed to explore the effectiveness of VR in comparison with RaD as suitable learning methods to build knowledge. In addition, we aimed to investigate students’ opinions with regard to task value and level of motivation when learning neuroanatomy by either RaD or VR. The authors hypothesized that students would develop a superior comprehension of the 3D relationship of the structures of the human brain when having worked with VR when compared to students who worked with RaD. Also, we believed that the students who worked with VR would be more engaged when learning neuroanatomy and that this would increase their level of motivation and positively influence experienced task value. The outcomes of this study can provide other educators with new insights how to effectively engage students when teaching neuroanatomy. Also, these outcomes will show teachers and students which technological innovations can be used to yield the greatest effect with regard to learning neuroanatomy.

## Materials and methods

### Ethical approval and participants

All methods carried out within this study were in agreement with the Statement on the Declaration of Helsinki and the Ethical Conduct of Clinical Studies. In addition, the study details were approved by Ethical Review Board of The Netherlands Association for Medical Education (NVMO) and registered (NERB dossier number 2018.8.2).

Students from the Faculty of Social Sciences at Radboud University were recruited to participate in this study. Students participated voluntarily and were recruited by using recruiting announcements, online advertisements and posters. Students signed up by sending an e-mail to one of the researchers (M.v.D.). Students who reported to have previously studied neuroanatomy were excluded due to the strong link between anatomy training and spatial ability (for reviews and meta-analyses see^52,53^). We deliberately chose to include students without an educational background in natural sciences, technology and mathematical reasoning as they are known to have inferior spatial and visual skills^54^. Also, this group of students represents a relatively understudied student population who might benefit from the innovations with regard to anatomy education as well as medical students^55^.

### Study design

**Figure 1:**
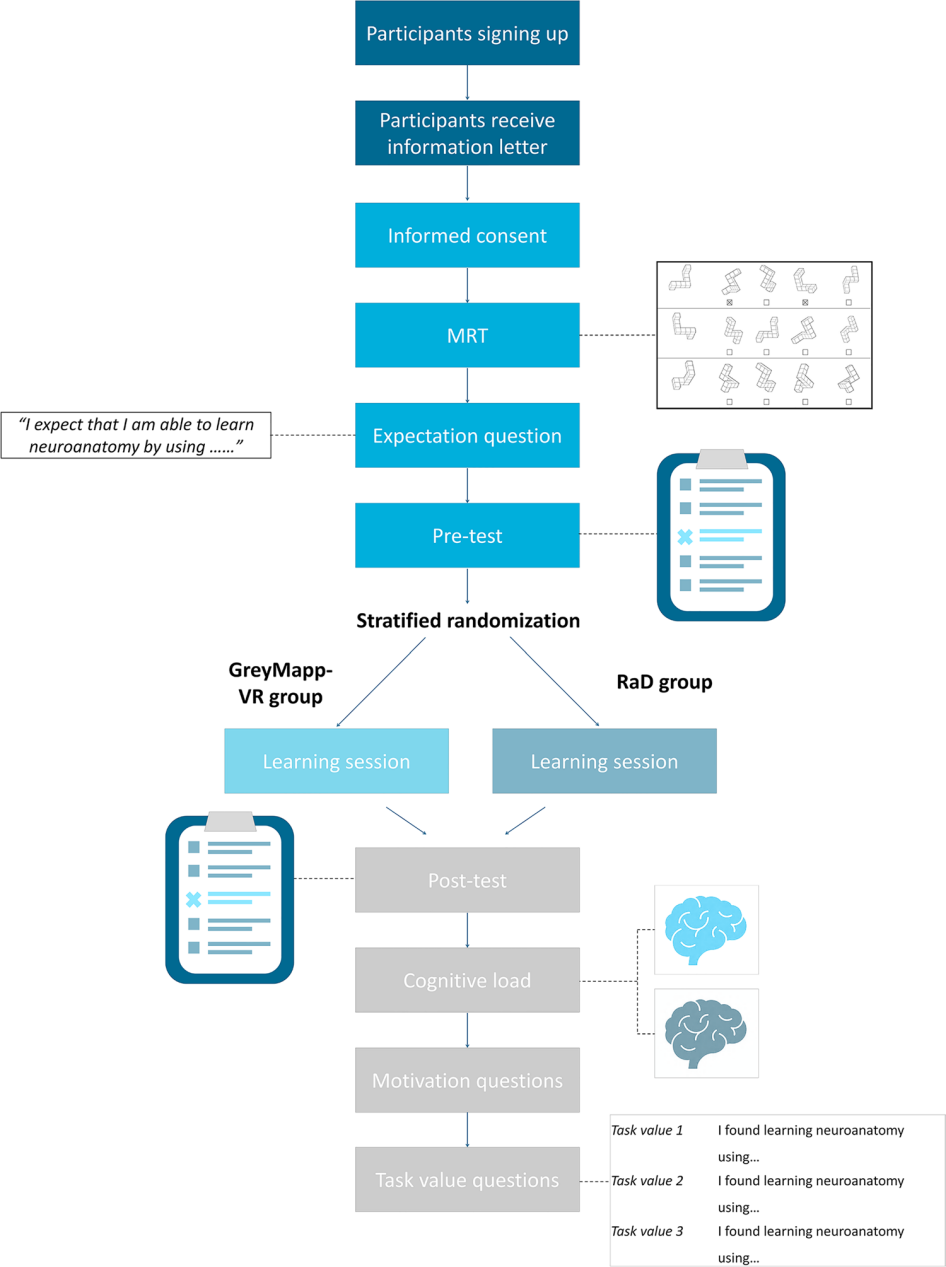
Schematic representation of the study design. *MRT* Mental rotation test, *VR* Virtual reality.

The study followed a pretest–posttest design. After signing the informed consent forms, participants were randomized over two groups. Randomization was carried out following the block randomization method to result in balanced groups with regard to number of included participants as well as with regard to gender^56^. Participants were informed which teaching tool they were going to work with prior to the pre-test. The first group used GreyMapp-VR to learn neuroanatomy (i.e., the VR-group). The second group learned neuroanatomy by using a laptop on which the annotated MRI data of GreyMapp was presented (i.e., the RaD-group). Participants started the experiment by taking the mental rotations test (MRT) to assess spatial abilities. Then, expectancy questions were answered by the participants, followed by a pre-test on neuroanatomy. Thereafter, students were given the opportunity to work with their randomly assigned learning method for a maximum of five minutes. When participants expressed that they understood the programs that they were supposed to work with, they received their assignments (on paper or within the VR environment) which they could use to study neuroanatomy in 30 min. The overall assignment was to study the structures’ names, their three dimensional characteristics and relationship to the surrounding structures. Participants were free to choose their learning strategy. After studying, a post-test similar to the pre-test was presented, which was to be followed by questions assessing task value and motivation (Fig. 1).

All participants were allowed to choose a preferred timeslot with the duration of 90 min to take part in the experiment. Each participant participated individually under the supervision of two of the investigators (M.v.D. and L.R.).

### Teaching applications

The present study uses an in-house-written application called GreyMapp-VR. GreyMapp was developed and first launched in 2016^57^. GreyMapp was designed with the aim of helping students master the 3D anatomy of the human brain in a modern and innovative way. An augmented reality version of GreyMapp has already been reported on before^15^ and is now freely available within the App Store (https://apps.apple.com/nl/app/greymapp/id1533656197?l=en).

GreyMapp-VR allows users to navigate a 3D brain model while being completely embedded in a VR-environment. The 3D model contains the ventricular system with the internal capsule and the major structures of the basal ganglia and the limbic system surrounding it. Source data of GreyMapp was derived from an annotated, 7 T post-mortem MRI scan of the human brain. The annotated source data was used for the RaD application in this experiment. Methods that were used to acquire the MR data and which were used to construct GreyMapp have been published by our group before^15,57^.

**Figure 2:**
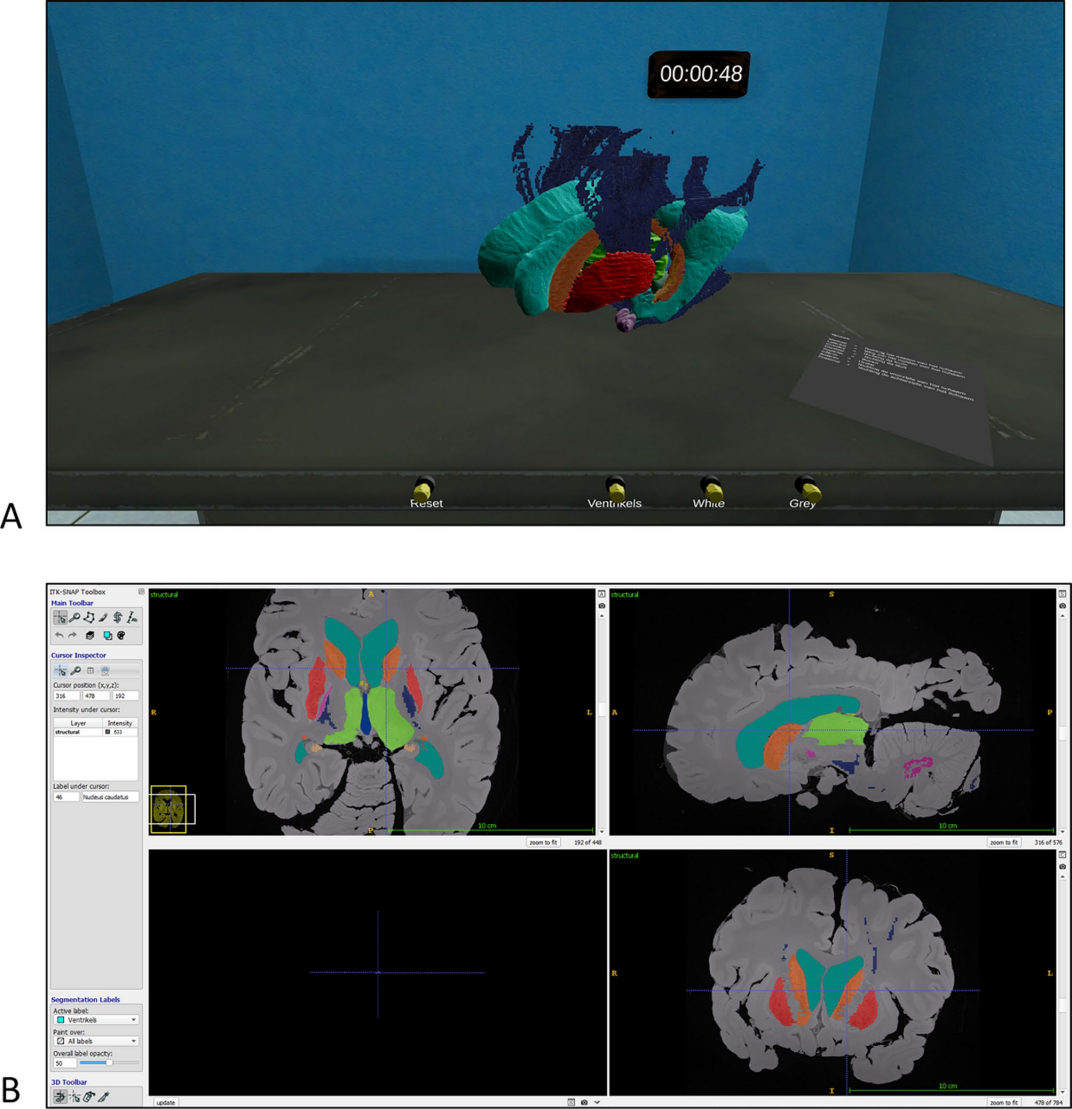
Exemplary images of the used teaching applications. (**A**) VR environment showing the 3D model in the VR environment (view on the brain model in the VR group. This figure shows the in-house created VR environment, constructed with Unity software package; https://unity.com). (**B**) Coronal, sagittal and coronal sections of the RaD environment (view on the screen in the control group; http://www.itksnap.org)^58^.

GreyMapp-VR was created by an affiliated software engineer using C# within Unity v 2018.4.5F (LTS) (*Unity Technologies ApS, San Francisco, CA*). Students were equipped with the HTC Vive Focus Plus (https://enterprise.vive.com/us/product/focus-plus/, accessed: 30-09-2020). The HTC Vive Focus Plus is a standalone VR headset with two controllers which support 6 degrees of freedom, indicating that rotation and spatial movements are tracked whilst using them. In the environment of GreyMapp-VR, the 3D model is positioned on a laboratory table. On the user’s right-handed-side, a cheat sheet could be found which helped participants to help remember anatomical orientations and -structures. Participants could walk around the VR-model, in addition to rotate the model 360 degrees using the controllers. The controllers could also be used to dismantle the VR-model and to point out different structures. When pointing at a structure, the name of the particular structure would appear. Figure 2A shows a screenshot of GreyMapp-VR.

The control group worked with the annotated MRI scan on which the GreyMapp-project is based. Images and annotations were shown by use of the software ITK-SNAP, which is a general-purpose interactive tool for image visualization^58^. ITK-SNAP provided an environment for visualizing complex 3D imaging data sets and offered linked visualization of two-dimensional image cross sections (i.e., sagittal-, coronal- and transversal planes). ITK SNAP allowed participants to work with MR data like radiologists by scrolling through the data using a cross-hair tool. In Fig. 2B, a screenshot of the built ITK-SNAP module is shown. Again, a cheat sheet could be found which helped participants to remember anatomical orientations and -structures.

Models used in GreyMapp-VR and in ITK SNAP were color-coded using red–green–blue-alpha (RGBA) codes that were identical for both teaching applications.

### Testing instruments and measures

The pre-test and the post-test were conducted to measure the knowledge of neuroanatomy. The content and format of the tests was constructed to reflect identical content. Pre- and posttest each consisted out of two parts. The first part comprised an extended matching test that included ten questions on spatial relations of different neuroanatomical structures. An example question is: ‘Which of the abovementioned structures is situated between the nucleus caudate and the fornix?’ (Correct answer: Lateral Ventricle). Maximum score for this part was 10 points. The second part of the tests was a multiple-choice test which included 20 questions (maximum score was 20 points). An example question is: ‘The capsula interna is situated medial/lateral from the putamen’ (Correct answer: Medial). Each question had two answer options. Tests were designed based on previous empirical experience and scientific papers produced by our group and others^11,15^. Based on Bloom’s taxonomy, the tests assessed learning information belonging to the Application Dimension. Learners were tested whether or not they were capable of implementing abstractions that were both similar and different from the learning situation^59^. None of the questions were accompanied by figures or pictures.

Pre-test and post-test were similar testing forms with a maximum score of 20 points each. Correction of guessing was carried out^60^ for the multiple choice questions to include choice weighting. Scores could not be lower than 0 points.

To assess variable spatial ability, the mental rotation test was used^61^, previously validated^62^ and redrawn by Peters and colleagues^63^. The MRT scores were used to assess whether significant differences existed in spatial ability. Students were awarded with a point when both of the stimulus figures that match the target figure were identified correctly. No credit was given for a single correct answer. Maximum MRT-score was 24 points.

The moderator expectancy value was measured with one question and aimed to measure the participants’ expectation on how well they thought they were going to perform using the teaching method. The question for the participants in the VR group was: ‘I expect that I am able to learn neuroanatomy by using Virtual Reality’. The question for the participants in the control group was: ‘I expect that I am able to learn neuroanatomy by using the RaD’. Answers were given on a 5-point Likert scale ranging from ‘I strongly disagree’ to ‘I strongly agree’. The moderator task value was measured with three questions and aimed to measure how the participants valued the teaching method that they used. The questions were extracted from the Motivated Strategies for Learning Questionnaire^64^ and adapted to fit this study. The answers provided an indication of how participants felt about the value of the teaching method by saying how interesting, fun and useful they found the teaching method (Table 1).

**Table 1:** Questions to measure the experienced task value.

Experience task value	
Task value 1	I found learning neuroanatomy using GreyMapp-VR/RaD interesting
Task value 2	I found learning neuroanatomy using GreyMapp-VR/RaD fun
Task value 3	I found learning neuroanatomy using GreyMapp-VR/RaD useful

Adapted from (Pintrich, 1991)^64^.

The covariable motivation was measured with one question to investigate how motivated participants felt when using their teaching method. This question was scored on a scale from 1 (not motivated) to 10 (very motivated). Cognitive load was assessed by use of a set of questions which have been previously investigated and reported^65,66^.

### Statistical analysis

The statistical package SPSS Statistics, version 26 (*IBM Corp., Armonk, NY*) was used for statistical analyses. The Cronbach’s alpha test was carried out to assess whether (1) the pre-test and post-test and (2) the experience task questions were internally consistent (i.e., assessing internal validity and reliability). In addition, McDonald’s omega coefficient was used to assess internal reliability of both tests as well. To use McDonald’s omega coefficient, the methodology as described by Hayes et al. and the accompanying software extension which can be integrated into SPSS Statistics was used^67^. Internal consistency is generally regarded acceptable when both Cronbach’s alpha value and McDonald’s omega coefficient was ≥ 0.7.

Statistics for the scores on the pre-test, post-test, mental rotation test, expectation-, task value- and motivation scores were calculated. Paired and unpaired student’s t-tests were applied to compare mean scores between the (1) pre-test results; (2) post-test results; (3) MRT scores; (4) expectation scores; (5) task value questions; (6) motivation scores; and (7) cognitive load questions between the GreyMapp-VR group and the control group. To analyze whether the GreyMapp-AR group and the control group had different distributions of categorical parameters (i.e., sex), a chi-squared test was conducted. Repeated-Measures Anova was done to analyze the effect of the different teaching methods. The variables expectation, task value and motivation were added to a Repeated Measures analysis with a covariable to investigate whether these had moderating effects. A Bonferroni post-hoc test was carried out to test for multiple comparisons. Furthermore, correlations between MRT score and post-test results were investigated by use of the Pearson Correlation Coefficient.

## Results

An overview of the included participants of this study is provided in Table 2. In total, 47 students with a mean age of 19.47 ± 0.54 years were included. Forty-three of the participants (91.5%) identified as female; four participants identified as male (8.5%). The majority of students (89.4%) studied Psychology; 6.4% of the participants studied Pedagogical Sciences. One student (2.1%) studied Sociological sciences and one student (2.1%) studied Law. The used anatomical tests and task value questions were found to be internally consistent with a Cronbach’s alpha of 0.803 and 0.814, respectively and a McDonald’s omega of 0.718 and 0.857, respectively. Corrected mean pre- and post-test scores, as well as other mean scores can be found in Table 2.

**Table 2:** Baseline characteristics per group and p-values of the inductive statistics.

Variable	Total group (n = 47)	GreyMapp-VR group (n = 23)	Control group (n = 24)	p-value	Cohen’s *d*
Age	19.47 ± 3.73	19.09 ± 4.58	19.83 ± 2.73	0.504	0.20
Gender	4 males 43 females	2 males 21 females	2 males 22 females	0.965	0.013
Study	42 psychology 3 pedagogical sciences 1 law 1 sociological sciences	20 psychology 1 pedagogical sciences 1 law 1 sociological sciences	22 psychology 2 pedagogical sciences	0.407	0.24
MRT	12.28 ± 2.71	12.70 ± 2.77	11.88 ± 2.64	0.304	0.30
Expectation	3.87 ± 0.61	3.70 ± 0.70	4.04 ± 0.46	0.019	0.58
Pre-test score	2.45 ± 3.27	2.57 ± 3.5	2.33 ± 3.10	0.900	0.04
Post-test score	12.94 ± 6.08	13.04 ± 5.99	12.83 ± 6.3	0.774	0.08
Cognitive load score	6.30 ± 1.50	6.09 ± 1.2	6.50 ± 1.75	0.352	0.30
Motivation	6.70 ± 1.92	7.78 ± 1.20	5.67 ± 1.93	< 0.001	1.31
Task value Interesting	4.06 ± 0.90	4.57 ± 0.51	3.58 ± 0.93	< 0.001	1.30
Task value fun	3.53 ± 1.16	4.26 ± 0.69	2.83 ± 1.09	< 0.001	1.56
Task value useful	4.06 ± 0.79	4.30 ± 0.56	3.8 ± 0.92	< 0.001	0.62

*MRT* Mental rotation test, *VR* Virtual reality.

When comparing the students in the GreyMapp-VR group versus the control group, no significant differences were found in gender-distribution (p = 0.965; Cohen’s d = 0.01; df = 1), study direction (p = 0.407; Cohen’s d = 0.24; df = 5) and distribution of age (p = 0.504; Cohen’s d = 0.20; F = 0.224; df = 45). No significant differences were found between the corrected pre- and post-test scores between groups (p = 0.900; Cohen’s d = 0.04; F = 0.043; df = 45 and p = 0.774; Cohen’s d = 0.08; F = 0.043; df = 45 respectively). No differences were found between groups with regard to mental rotation test (p = 0.913; Cohen’s d = 0.30; F = 0.012; df = 45).

With regard to expectancy, the RaD group showed to have a significantly higher score than the students in the VR group (p = 0.019; Cohen’s d = 0.58; F = 5.972; df = 45). Task value scores regarding finding a task interesting, useful and fun were found to be significantly different (all p < 0.001; Cohen’s d = 1.30; F = 4.567; df = 45, Cohen’s d = 0.62; F = 0.553; df = 45 and Cohen’s d = 1.56; F = 7.053; df = 45 respectively) in favor of the GreyMapp-VR group. Motivation was also found significantly different between groups in favor of the VR group (p < 0.001; Cohen’s d = 1.31; F = 3.473; df = 45). Cognitive load scores were found not to be different between groups (p = 0.352; Cohen’s d = 0.30; F = 0.645; df = 45).

Repeated measures ANOVA showed a significant improvement in test results when comparing the pre-test and post-test results (p < 0.0001; Partial Eta Squared = 0.766; F = 158.76; df = 47). Significant differences were found when comparing pre-test and post-test results in the VR-group (p < 0.0001; Partial Eta Squared = 0.749; F = 109.24; df = 22) and control-groups (p < 0.0001; Partial Eta Squared = 0.783; F = 85.52; df = 23). No significant differences were found in improvement of test scores between groups (p = 0.408; Partial Eta Squared = 0.546; F = 0.70; df = 47).

To analyse the moderating effect of expectancy, task value and motivation on learning outcomes, a Repeated Measures ANOVA with covariates was performed. These analyses showed that motivation and expectancy did not moderate this relationship (p = 0.981; Partial Eta Squared < 0.0001; F = 0.001; df = 44 and p = 0.970; Partial Eta Squared < 0.0001; F = 0.001; df = 44, respectively). Task value, however, was found to significantly moderate this relationship (p = 0.001; Partial Eta Squared = 0.070; F = 3.40; df = 45). Sub analysis showed that task value for finding the methods interesting and useful were significantly impacting the learning effects (p = 0.025; Partial Eta Squared = 0.112; F = 5.43; df = 43 and p = 0.008; Partial Eta Squared = 0.065; F = 3.01; df = 44, respectively) in favour of the VR group. Task value for experiencing the methods as fun or entertaining were not significantly impacting the learning outcomes (p = 0.527; Partial Eta Squared = 0.407; F = 13.74; df = 44). MRT scores and post-test scores were not correlated (r = 0.179; p = 0.228).

## Discussion

This study showed that VR and RaD are two effective methods to help students learn neuroanatomy, which is in line with the multimodal learning theory^46^ as both VR and RaD are considered interactive learning environments suitable for knowledge building. In terms of knowledge building there were no differences between the methods which can be explained that both methods were comparable in that the verbal representations and non-verbal representations were meaningful, closely related in content (coherence principle) and in space and time (contiguity principle). Both methods offer interactivity and although VR offers more opportunity to interact with the anatomical structures, this did not seem to add value to the results.

Although it is known that student’s spatial ability influences anatomy learning^68^, this study did not find a significant relationship between the learning effect and spatial ability as measured by MRT. Other papers reported that instruction with desktop rendering was more effective than identical instruction with VR because the information in the VR environment created more overload in working memory. This overload in turn distracted the learners from the essential content^44,48,69^. However, these findings were not corroborated by the current study.

Other factors for effective learning (i.e., expectancy, motivation and task value) were also included in this study. Prior to the practical work with the teaching methods, there was a significant difference between the expectancy rates for students in favor of the RaD group. This is important for learning due to the fact that when individuals expect that they are going to be successful in performing a task, they will use more advanced cognitive strategies and be more persistent when performing this task^51^. However, why expectancy rates were significantly higher for students working with RaD remains partially elusive. A possible explanation might be that the included participants felt more comfortable with RaD displayed on a computer screen than with VR models wearing a VR headset. It is known that junior doctors find working with radiological data exciting when these data are of sufficient quality^70^. Whether this also accounts for non-medical students remains unclear. However, after having worked with either VR or RaD, it was found that VR was valued more as a task for learning neuroanatomy than using the screen-based RaD assignment. It was also found that the learning effects were significantly impacted by task value scores regarding finding the learning methods interesting, fun and useful, favoring students working with VR. This could be explained by the fact that VR is a relatively new and exciting teaching method (i.e., the novelty effect). In addition, the novelty effect, which is defined as “a person’s subjective first response to (using) a technological innovation”, plays an important role in the studies that used technological innovations to teach anatomy^71^. Previous studies noted that as the novelty effect wears off, users discontinue their use of new technologies, indicating a loss of interest^71,72^. To find out whether or not the task value will be persistent during a longer period of time, further research is necessary. With regard to motivation scores, a significant difference was found in favor of the VR group. In general, when a task is valued more, this has a positive effect on the motivation of students^73^. These insights might be important when searching for fitting hands-on neuroanatomy learning tools.

The results from the present study are in agreement with findings of previous studies^29,44,49^. The present results partially conflict with the results from Kurul et al. (2020) as they found that students in the VR group significantly improved more than students who learned anatomy by attending a presentation on the subject. These results suggest that VR systems can be used as an alternative method to anatomy lectures^30^. An interesting difference in the design of Kurul et al. and the here presented design concerns that the groups of Kurul et al. compared an interactive multimodal learning environment (i.e., VR) with a non-interactive multimodal learning environment (i.e., presentation). Therefore, the circumstances are different between groups, which should be considered a confounding factor in this study. On the other hand, the study of Kurul et al. used learning strategy as assessed by the Kolb Learning Style Inventory for stratification purposes. The Kolb Learning Style Inventory was developed by David Kolb in 1976 and divides learning strategies into four stages/categories of learning: diverger, assimilator, converger, and accommodator^74^. It has already been suggested that these learning styles are important when learning with VR^75^. More specifically, learning with VR showed to elicit significant positive effects for both assimilator learners and accommodator learners^75^. In addition, both studies report that, during the lessons, VR participants were more likely to exhibit adverse effects such as headaches, dizziness or blurred vision^29,30^. These adverse events were not reported in the current study.

One of the limitations of the present study concerns the relatively small sample size which complicates the generalization of the statements made in this study. In addition, the population is skewed with regard to gender of the participants. According to a review by Severiens and Ten Dam^76^, no differences in learning styles were found in the dimensions of Kolb’s learning preferences only that men showed a greater preference than women for the assimilator and converger learning styles. This indicated that men preferred learning by a concise, logical approach as well as using problem solving as a learning style (i.e., the abstract conceptualization mode of learning)^76^. Another limitation concerns the lack of a third group in which students were taught neuroanatomy in the traditional fashion. Adding this group to the study protocol would have made it possible to investigate the differences between learning outcomes when studying with the traditional study materials as compared to studying with the here presented interactive multimodal learning environments. The lack of this group, however, has no considerable impact on the conclusions drawn in this paper. Furthermore, pre-test scores were very low which could be regarded as a limitation and a strength. With such low pre-test scores, a significant improvement in anatomical test scores is more plausible. On the other hand, as students had very limited knowledge on the subject prior to the experiment, it could be concluded that the exclusion criteria were adhered to. Major strengths of this paper concern the fact that various covariates were assessed, including spatial ability, expectancy and task value. The covariate “motivation” has been investigated by use of a pragmatic question since no validated instrument exists to test level of motivation when students work with VR features or other interactive multimodal learning tools. In addition, the relative robustness of the other used psychometric instruments is a relative drawback of this type of research.

Although this study has some limitations, the recent study also has some implications for future research. It has now been shown that students experience a higher task value using VR, suggesting that they find VR more motivating and interesting, fun and useful than learning using the RaD. However, whether this effect persists on the long-term remains unknown.

## Conclusion

We showed that both VR and RaD are effective interactive multimodal learning tools for teaching non-medical students neuroanatomy. We found no significantly different test scores between groups of students working with VR and RaD. It was, however, observed that GreyMapp-VR motivated students more to study neuroanatomy following the mechanisms as proposed by the expectancy-value theory. Therefore, this study opens doors to help implement the use of VR in neuroanatomy education. However, whether these effect remain present on the long-term remains elusive.
